# Unveiling the novel value of TREM1 in the proneural-mesenchymal transition of glioma via tumor-associated macrophages

**DOI:** 10.3389/fimmu.2025.1662351

**Published:** 2025-11-27

**Authors:** Chao Zhang, Ben Hu, Chao Wang, Shiqiang Hou, Ning Lin

**Affiliations:** 1 Department of Neurosurgery, The Affiliated Chuzhou Hospital of Anhui Medical University, The First People’s Hospital of Chuzhou, Chuzhou, China; 2 Science and Technology Innovation Center, Guangzhou University of Chinese Medicine, Guangzhou, China

**Keywords:** TREM1, glioblastoma, EMT, proneural-to-mesenchymal transition, tumor-associated macrophages

## Abstract

**Background:**

Tumor-associated macrophages (TAMs) constitute the most abundant immune cell population in the glioblastoma (GBM) tumor microenvironment. These macrophages critically influence multiple pathological processes in GBM, including recurrence, drug resistance, and immune evasion. However, the role of TREM1 in mediating proneural-to-mesenchymal transition (PMT) in TAMs remains unclear.

**Methods:**

Single-cell RNA sequencing analysis of 24,366 cells from seven GBM patients identified nine distinct cellular populations, including TAMs and malignant cells. Deconvolution analysis of TCGA mRNA-seq data complemented these findings. Differential expression analysis, weighted gene co-expression network analysis, and LASSO-Cox regression consistently identified TREM1 in TAMs as a PMT-associated biomarker. A co-culture system demonstrated TREM1’s influence on GBM cell malignancy and PMT progression *in vitro*, while intraperitoneal LP17 administration assessed its impact on tumor growth.

**Results:**

High TAM infiltration correlated significantly with poor clinical outcomes in GBM patients. Integrated scRNA-seq and mRNA-seq analyses established TREM1 in TAMs as a key PMT biomarker. LP17-mediated TREM1 inhibition attenuated PMT through modulation of the TLR4/PI3K/AKT/mTOR pathway and reduced tumor growth *in vivo*.

**Conclusion:**

TREM1 functions as an oncogenic biomarker in TAMs that drives GBM PMT via the TLR4/PI3K/AKT/mTOR axis, with inhibition of TREM1 demonstrating therapeutic potential in both ex vivo and *in vivo* models.

## Graphical Abstract

**Figure:**
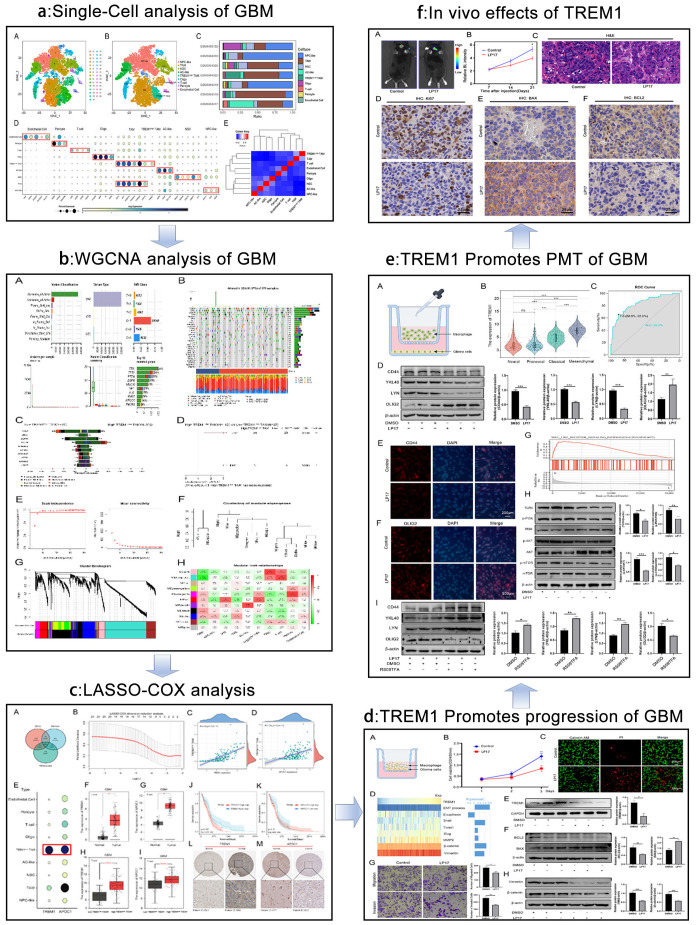
**(a)** Identification of TREM1 as a PMT Biomarker; **(b)** WGCNA analysis of GBM; **(c)** LASSO-COX analysis; **(d)** TREM1 Promotes progression of GBM; **(e)** TREM1 Promotes PMT of GBM; **(f)***In vivo* effects of TREM1.

## 1 Introduction

Glioblastoma (GBM) remains the most aggressive and lethal primary intracranial tumor, exhibiting pronounced inter- and intratumoral heterogeneity (1). Current standard therapies involving surgical resection followed by radiotherapy fail to substantially improve patient survival (2). The tumor microenvironment (TME) comprises not only neoplastic cells but also astrocytes, tumor-associated macrophages (TAMs), and oligodendrocytes, which collectively influence disease progression (3, 4). Emerging evidence highlights the critical involvement of tumor-infiltrating immune cells in mediating immunosuppression while actively modulating tumor growth, metastasis, and therapeutic resistance (5). Among these, TAMs represent the predominant immune population in GBM and significantly influence both tumor progression and treatment response (6). These macrophages correlate with unfavorable prognosis across multiple malignancies by impairing antitumor immunity, enhancing angiogenesis, and facilitating invasive growth (7–9). GBM demonstrates four molecular subtypes-neural, proneural, classical, and mesenchymal-with the proneural-to-mesenchymal transition driving acquisition of the mesenchymal phenotype (10). This phenotypic shift contributes to therapeutic resistance and ultimately worsens clinical outcomes in GBM patients (11).

The advent of single-cell sequencing has enabled detailed exploration of tumor microenvironment heterogeneity, facilitated the construction of single-cell atlases, and allowed precise assessment of cellular and molecular states (12). This approach addresses the limitations of bulk RNA-seq in resolving cellular subtypes, while computational analysis provides deeper insights into how both intrinsic and extrinsic cellular factors influence tumor biology (12). scRNA-seq analysis of GBM specimens previously identified four malignant cell subtypes: neural progenitor-like (NPC-like), oligodendrocyte progenitor-like (OPC-like), astrocyte-like (AC-like), and mesenchymal-like (MES-like), with MES-like cells demonstrating the most aggressive phenotype (13). These MES-like tumor cells show strong associations with macrophage infiltration, and NF1 mutations or deletions that enrich for MES-like populations further promote macrophage recruitment (14). However, these discoveries have yet to translate into significant therapeutic breakthroughs for GBM.

Triggering Receptor Expressed on Myeloid Cell-1 (TREM1/CD354) belongs to the TREM family and regulates dendritic cell and macrophage activation (15–17). This receptor shapes the immunosuppressive tumor microenvironment through cytokine production, T cell differentiation, and hepatocellular carcinoma development (18, 19). Clinical studies associate increased TREM1 expression with advanced lung, liver, and pancreatic cancers, where it predicts poor outcomes. In prostate cancer, androgen receptor signaling in macrophages drives TREM1-mediated tumor progression (20). While these findings establish TREM1’s oncogenic functions, its precise mechanisms in glioma-associated macrophages-particularly regarding immunosuppression and proneural-to-mesenchymal transition in glioblastoma-require further investigation.

The role of TREM1 in TAMs within the immunosuppressive TME of gliomas remains poorly understood. Recent advances in genomic databases now enable systematic investigation of TREM1’s biological functions through integrated single-cell RNA sequencing and transcriptomic analysis in GBM patients. Using bioinformatics tools to analyze single-cell sequencing data from GBM cohorts, we found TAMs represent the dominant immune cell population, with infiltration levels strongly predicting clinical outcomes. Lasso-Cox regression analysis further pinpointed TREM1 as a candidate therapeutic target. TREM1 shows significant enrichment in aggressive GBM subtypes and its inhibition functionally mediates anti-tumor effects by suppressing the glioma proneural-to-mesenchymal transition through modulation of the TLR4/PI3K/AKT/mTOR pathway. These findings position TREM1 as a potential target for refining immunotherapy approaches in glioma.

## 2 Methods

### 2.1 Data acquisition and download

The scRNA-seq data for GBM were obtained from the Gene Expression Omnibus (GEO: https://www.ncbi.nlm.nih.gov/geo/) under accession number GSE135045. This dataset comprises single-cell data from 7 GBM patient specimens, from which a total of 24,366 high-quality cells passed our quality control filters and were used for downstream analysis. The corresponding bulk mRNA-seq data, clinical annotations, and SNV profiles for GBM were acquired from The Cancer Genome Atlas (TCGA: http://portal.gdc.cancer.gov/) GBM project via the Genomic Data Commons Portal.

### 2.2 Cell culture and treatments

LN229 cells were maintained in Dulbecco’s Modified Eagle’s Medium (DMEM) supplemented with 10% fetal bovine serum (FBS) at 37°C in a 5% CO_2_ humidified atmosphere. THP-1 cells were grown in RPMI-1640 medium containing 0.05 mM β-mercaptoethanol. THP-1 cells were first differentiated into M0 macrophages by pretreatment with 100 ng/mL PMA for 48 hours, and were then polarized to the M2 phenotype by further treatment with 20 ng/mL IL-4 for an additional 48 hours. The TREM1 inhibitory peptide LP17 (HY-P3400) and TLR4 agonist RS09TFA(GC63760) were obtained from MedChemExpress (MCE) and GLPBIO, respectively. Both compounds were dissolved in dimethyl sulfoxide (DMSO) for stock solution preparation.

### 2.3 scRNA-Seq data processing and analysis

Single-cell RNA sequencing data underwent standard quality control filtering before analysis. The following stringent thresholds were applied to ensure the inclusion of high-quality cells and the exclusion of technical artifacts: (1) Cells were retained only if the total UMI count (nCount_RNA) was greater than or equal to 1,000. (2) The number of unique genes detected per cell (nFeature_RNA) was required to be between 200 and 10,000. (3) Cells with a mitochondrial gene percentage (percent.mt) exceeding 20% were excluded to remove apoptotic or damaged cells. (4) Cells with a ribosomal gene percentage (percent.rb) exceeding 20% were also excluded. After applying these criteria, 24,366 high-quality single cells from seven samples remained for downstream analysis.

The scRNA-seq data were processed in R 4.4.0 using the Seurat package (v.4.4.0) (21), where normalization was performed with the LogNormalize method. Highly variable genes were selected via the FindVariableFeatures function, followed by batch effect correction using Harmony (22). We determined the optimal number of principal components through JackStraw analysis and performed dimensionality reduction by applying PCA to the top 1500 highly variable genes. Subsequent clustering analysis with FindNeighbors and FindClusters (resolution=1.5) yielded 29 distinct cell populations. Cluster-specific differentially expressed genes were identified using the Wilcoxon rank sum test implemented in FindAllMarkers (cutoff: min.pct=0.25, logfc.threshold=0.25).

### 2.4 Deconvolution of cells in different states from single-cell data

The “BayesPrism” R package deconvoluted diverse cell states using a Bayesian framework (23). This approach analyzed the cellular composition of distinct clusters in each scRNA-seq sample by leveraging single-cell resolution features. Mitochondrial and ribosomal genes, which lacked relevance, were excluded to refine the deconvolution. Protein-coding genes with the highest cross-assay consistency were prioritized for reconstruction. By treating scRNA-seq data as prior information, BayesPrism jointly inferred the posterior distribution of both cell type proportions and gene expression profiles from bulk RNA-seq data.

### 2.5 WGCNA analysis

Weighted Gene Co-expression Network Analysis (WGCNA) provides a systems biology framework for characterizing gene interaction patterns across transcriptomic datasets. We applied the “WGCNA” R package to build a co-expression network using differential gene expression profiles from the TCGA-GBM cohort, revealing both coordinated expression modules and clinically relevant biomarker genes through their association with phenotypic traits. The “pickSoftThreshold” function determined the optimal soft threshold (power) for network construction. The adjacency matrix (aij) was then calculated as aij = |Sij|β, where Sij denotes the Pearson correlation-based similarity matrix for all gene pairs and β represents the selected soft power value. This transformation produced a weighted correlation matrix that captured gene-gene interaction strengths. Hierarchical clustering of the topological overlap matrix (1-TOM) subsequently identified highly correlated modules that were prioritized for clinical correlation analysis.

### 2.6 LASSO-COX dimension reduction analysis

This study employed the LASSO-COX algorithm via the “glmnet” R package to analyze survival time, status, and gene expression data. The model was optimized by selecting λ-lse as the most suitable regularization parameter. Analysis of GBM patient overall survival data revealed two candidate genes with their associated λ values.

### 2.7 Functional enrichment analysis

To examine TREM1’s functional role, we analyzed genes most strongly correlated with TREM1 (Pearson R > 0.60, p < 0.01) through functional enrichment. These genes underwent GO and KEGG pathway analysis via the DAVID database (https://david.ncifcrf.gov/), using official gene symbols with Homo sapiens as the designated species.

### 2.8 Gene set enrichment analysis

The GSEA software was downloaded from the official GSEA (GSEA: https://www.gsea-msigdb.org/gsea/index.jsp/) website. Samples were stratified into two groups according to their calculated risk scores. We selected the c2.cp.kegg.v7.4.symbols.gmt gene set collection from the Molecular Signatures Database for analysis. The resampling procedure employed 1,000 permutations with gene set size thresholds set between 5 and 5,000 genes. The analysis primarily relied on gene expression profiles and risk group classifications.

### 2.9 Western blot analysis

Proteins were extracted with RIPA buffer and separated by electrophoresis on a concentrated gel before transfer to a PVDF membrane. The membrane was blocked for 2 hours at room temperature with TBST containing 1.5% milk to reduce nonspecific binding. After blocking, it was incubated overnight at 4 °C with the primary antibody. Three TBST washes removed unbound primary antibody, followed by a 2-hour incubation with the secondary antibody at room temperature. Protein bands were visualized after a final TBST wash by applying developer solution to the membrane. The primary antibodies used were as follows: TREM1 (ZEN-BIOSCIENCE), TLR4 (ZEN-BIOSCIENCE), p-PI3K (Proteintech Group, Inc.), PI3K (Proteintech Group, Inc.), p-AKT (Proteintech Group, Inc.), AKT (Proteintech Group, Inc.), p-mTOR (Proteintech Group, Inc.), mTOR (Proteintech Group, Inc.), BAX (Proteintech Group, Inc.), BCL2 (Proteintech Group, Inc.), Vimentin (Proteintech Group, Inc.), β-catenin (Proteintech Group, Inc.), CD44 (Wuhan Fine Biotech Co., Ltd.), YKL40 (Wuhan Fine Biotech Co., Ltd.), LYN (Wuhan Fine Biotech Co., Ltd.), OLIG2 (Wuhan Fine Biotech Co., Ltd.), and GAPDH (Proteintech Group, Inc.).

### 2.10 Transwell invasion and migration assays

Cell migration and invasion were evaluated with Transwell assays. For migration assays, LN229 and THP-1 cells suspended in 200 μL serum-free RPMI-1640 medium were seeded into uncoated upper chambers (Wuhan Servicebio Technology CO.), while 800 μL complete DMEM medium was added to the lower chambers of 24-well plates. Invasion assays followed the same protocol but employed Matrigel-coated chambers. LP17 was administered to cells in the upper chambers during both experimental conditions.

Following incubation, cells were gently removed from the chamber’s inner surface using a cotton swab. The cells were then fixed with 4% paraformaldehyde (PFA) for 15 minutes at room temperature. After three PBS washes, fixed cells were stained with 0.1% crystal violet solution for 30 minutes. The chambers were rinsed with PBS and mounted on glass slides. Stained cells were visualized and imaged using an inverted microscope.

### 2.11 Cell viability assay

Cell viability was measured with the Cell Counting Kit-8 (CCK-8) assay. LN229 cells were seeded in the lower chamber of a 24-well Transwell plate, with THP-1 cells placed in the upper chamber and treated with LP17. After incubation, CCK-8 reagent was added to each well following the manufacturer’s instructions. The plates were incubated at 37°C for 2 hours before measuring absorbance at 450 nm.

### 2.12 Calcein/propidium iodide staining

Live and dead cells was assessed using the AM/PI kit. Cells were seeded in 24-well plates and treated as per experimental conditions. After washing with PBS, 200 µL of AM/PI working solution was added, followed by incubation at 37°C for 30 min. Cells were then observed and imaged using a fluorescence microscope.

### 2.13 Immunofluorescence staining

After removing the cell culture medium, we washed the cells with PBS and fixed them in 4% formaldehyde for 15 minutes. Subsequent PBS washes preceded permeabilization with 0.5% Triton X-100 for 30 minutes, followed by a 30-minute blocking step using 5% goat serum. Primary antibody incubation proceeded overnight at 4°C. The following antibodies were used: CD44 (Wuhan Fine Biotech Co., Ltd.) and OLIG2 (Wuhan Fine Biotech Co., Ltd.). The next day, PBS washes were performed before a 2-hour incubation with fluorescent secondary antibody. Following final PBS washes, DAPI staining for 5 minutes enabled fluorescence microscopy visualization.

### 2.14 *In vivo* glioma model construction

To establish an *in vivo* glioma model, we stereotaxically implanted luciferase-expressing GL261-Luc cells into the right striatum of C57BL/6 mice. After standard surgical preparation, a midline scalp incision exposed the skull, and a 1-mm burr hole was drilled 2.0 mm lateral to the sagittal suture and 0.5 mm posterior to the coronal suture. A micro-syringe containing the cell suspension was vertically inserted through the craniotomy to a depth of 3 mm and then retracted 1 mm prior to injection. A total of 5×10^5 GL261-Luc cells in 5 μL of PBS were infused at 1 μL/min, with the needle remaining in place for 5 minutes post-injection before slow withdrawal. Bone wax sealed the craniotomy, followed by wound closure with sutures and prophylactic antibiotic administration. For therapeutic intervention, mice in the LP17 treatment group received intraperitoneal injections of the TREM1 inhibitory peptide LP17 at a dosage of 1 mg/kg every two days, starting from day 7 post-inoculation until the end of the study. At the experimental endpoint (day 21 post-modeling), bioluminescence imaging was performed using the AniView imaging system (Biolight Biotechnology).

### 2.15 Statistical analysis

Data analysis and graphical representations were performed using R software and GraphPad Prism. Statistical comparisons between two groups were performed using two-tailed Student’s t-tests. For comparisons among more than two groups, one-way analysis of variance (ANOVA) was employed, followed by Tukey’s *post hoc* test for multiple comparisons. The log-rank test was specifically used to assess the statistical significance of differences in survival curves from the Kaplan-Meier analysis. A p-value of less than 0.05 was considered statistically significant in all analyses. *p < 0.05, **p < 0.01, ***p < 0.001.

## 3 Result

### 3.1 GBM single-cell mapping analysis

**Figure 1:**
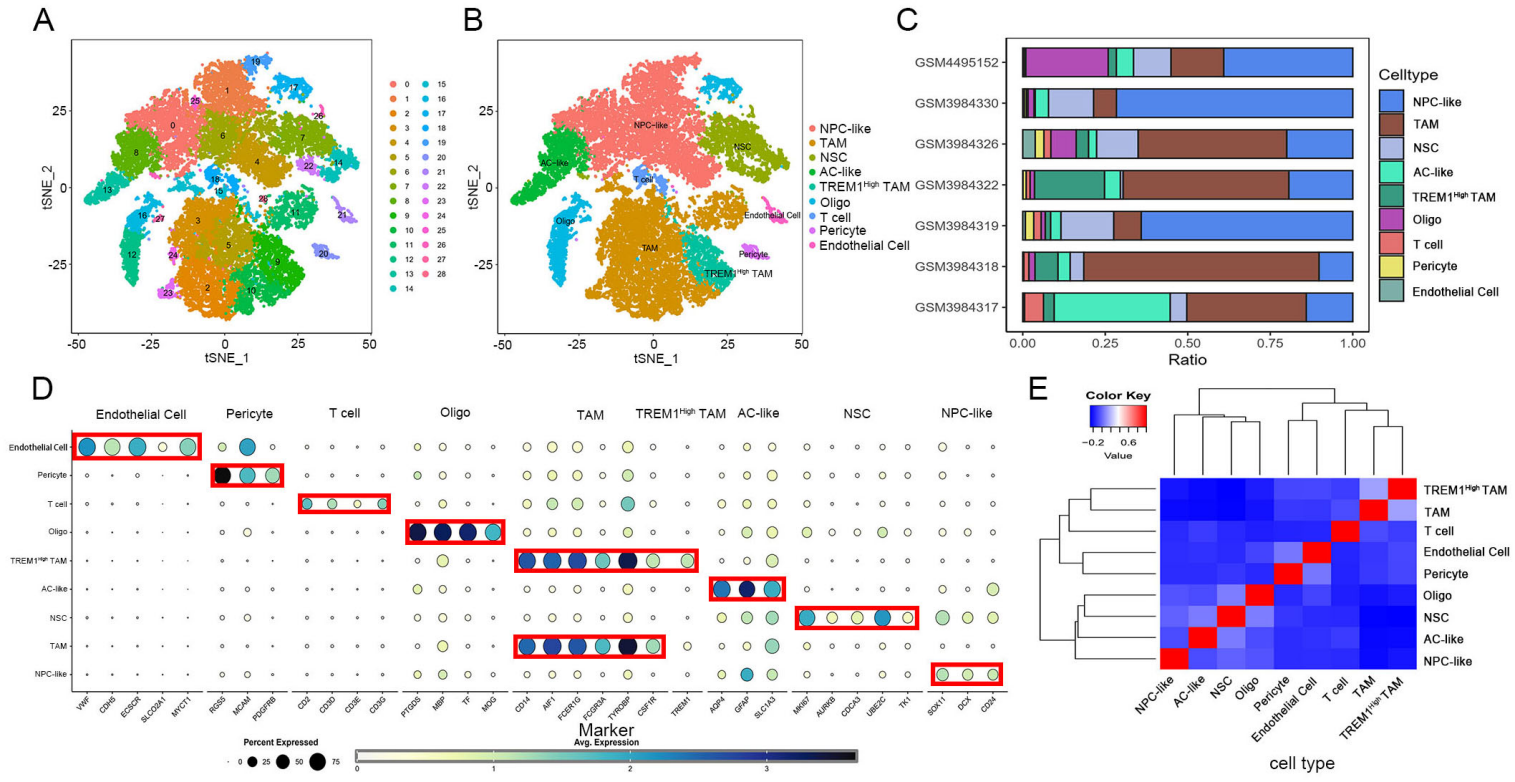
GBM Single-Cell Mapping. **(A)** Application of the t-SNE algorithm to visualize and cluster the single-cell sequencing data, leading to the identification of 29 distinct cellular subclusters; **(B)** Utilization of the t-SNE algorithm to delineate nine cellular subclusters, based on the expression levels of marker genes; **(C)** Bar graphs presenting the composition ratios of various cellular subclusters among different patients; **(D)** Dot plots depicting the expression levels of marker genes within designated cellular clusters; **(E)** Conducted correlation analyses between the different cellular subclusters.

To characterize cellular heterogeneity within the TME of GBM, we performed scRNA-seq on seven patient specimens. After applying quality control thresholds for gene detection per cell, we retained 24,366 cells while excluding those with either insufficient or abnormally high gene counts (**Supplementary Figure S1**). Data normalization and Harmony integration addressed inter-sample variability. Using the Seurat pipeline, we performed dimensionality reduction and unsupervised clustering to visualize patient-specific cell distributions before and after batch correction. The ElbowPlot function guided dimensionality selection across different reduction methods (Harmony or PCA), with 10 principal components retained for downstream analysis. This approach identified 29 discrete cell clusters defined by differentially expressed genes (**Figure 1A**).

By analyzing cell populations annotated with characteristic marker genes and visualizing the data through t-SNE, we classified cells expressing PTPRZ1, NES, EGFR, SOX2, and GFAP as malignant cells (24). The tumor microenvironment in GBM patients primarily consisted of malignant cells, TAMs, T cells, endothelial cells, pericytes, neural stem cells, and normal oligodendrocytes, with these subtypes consistently observed across all seven patients (**Figures 1B, C**). Published WES data (25) revealed that malignant cells exhibited both AC-like (marked by AQP4, GFAP, and SLC1A3) and NPC-like (marked by SOX11, DCX, and CD24) phenotypes, though distinguishing MES-like from OPC-like malignant cells proved difficult in our dataset. Non-tumor components included endothelial cells (expressing VWF, CDH5, ECSCR, SLCO2A1, and MYCT1), pericytes (marked by RGS5, MCAM, and PDGFRB), normal oligodendrocytes (showing PTGDS, MBP, TF, and MOG expression), and TAMs (identified by CD14, AIF1, FCER1G, FCGR3A, TYROBP, and CSF1R) (**Figure 1D**). Clustering analysis uncovered a unique TAM subset characterized by exclusive TREM1 expression, which we designated as TREM1^High^ TAMs. Patient-specific variation in cell type proportions-particularly the diverse states of tumor cells-reinforced GBM’s hallmark heterogeneity, with TAMs representing the most prevalent population (**Figure 1C**). The correlation matrix demonstrated relative independence among the nine identified cell subpopulations, validating our annotation approach (**Figure 1E**).

### 3.2 The GBM convoluted cell population, TREM1^High^ TAM, is strongly associated with immunity and inflammation

GO-based functional analysis showed significant enrichment of TREM1^High^ TAM cell clusters-linked to worse GBM prognosis-in biological processes like signal transduction, inflammatory response, apoptotic process, immune response, and angiogenesis (**Supplementary Figure S2A**). These findings imply that inflammatory and immune mechanisms driven by TREM1^High^ TAMs may contribute to GBM progression. Cellular component analysis highlighted associations with membrane structures, including the plasma membrane and cytosol (**Supplementary Figure S2B**), while molecular function analysis identified protein binding as the predominant activity (**Supplementary Figure S2C**). KEGG and GSEA analyses further connected poor GBM prognosis to TNF, NF-κB, and PI3K/AKT signaling pathways (**Supplementary Figure S2D**), with elevated activity observed in patients exhibiting high TREM1^High^ TAM scores (**Supplementary Figure S2E**). GSVA of 7,658 biological functions revealed strong correlations between high-scoring TREM1^High^ TAM groups and immune cell chemotaxis/activation (**Supplementary Figure S3**). Pearson correlation analysis of TREM1 co-expressed genes yielded consistent results (**Supplementary Figure S4**). Collectively, these data implicate immune and inflammatory pathways as key mediators of TREM1^High^ TAM-associated GBM aggressiveness.

### 3.3 SNV and WGCNA analysis of the GBM convoluted cell population

**Figure 2:**
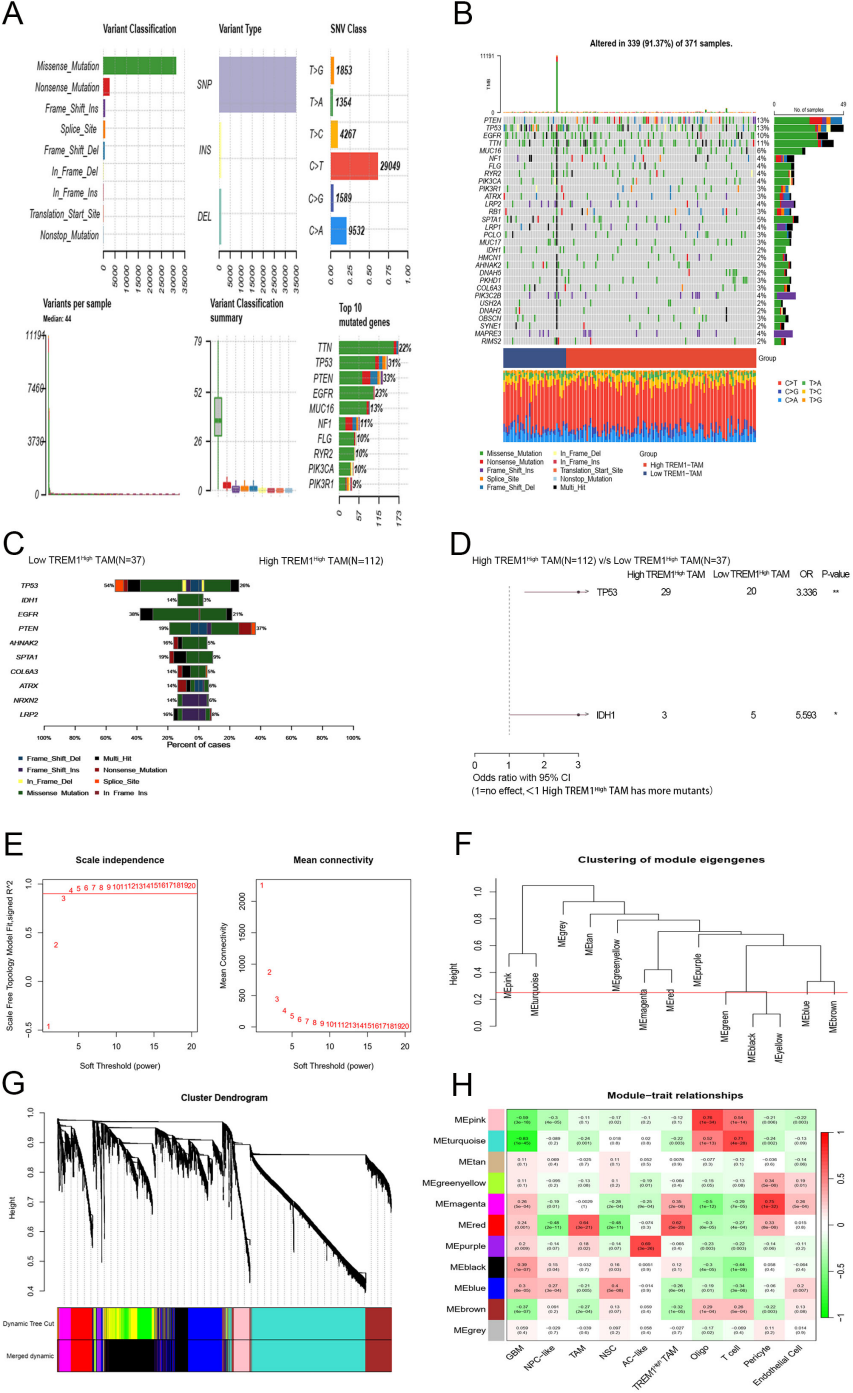
SNV and WGCNA Analyses in GBM Patients. **(A)** Missense mutations, SNPs, and C>T transitions are prevalent in GBM mutations; **(B)** A waterfall plot illustrating the 30 most frequently mutated genes across GBM samples; **(C)**. A list of the most frequently altered genes in the various TREM1^High^ TAM subgroups; **(D)**. TP53 and IDH1 mutations are more frequently observed in the High TREM1High TAM group; **(E)** Scale-free fit indices for soft threshold powers. The soft threshold power (β) in WGCNA was determined based on the scale-free R2 (R2 = 0.90). The left panel depicts the relationship between β and R2, while the right panel illustrates the relationship between β and average connectivity; **(F)** Clustering of module-trait genes; **(G)** Cluster dendrogram of the coexpression network modules; **(H)** Correlation analysis between characterized genes and clinical traits.

We analyzed genomic characteristics in both clusters using SNV data from the TCGA dataset. Missense mutations, SNPs, and C>T transitions dominated the mutational profile of GBM patients. The most frequently mutated genes—TTN, TP53, PTEN, EGFR, and MUC16—highlighted the heterogeneity of TREM1^High^ TAM populations in GBM (**Figure 2A**). The waterfall plot revealed patient-specific mutation patterns (**Figure 2B**), with distinct mutation frequency distributions across TREM1^High^ TAM subgroups (**Figure 2C**). The high TREM1^High^ TAM group exhibited particularly elevated TP53 and IDH1 mutation rates (**Figure 2D**).

To identify key modules associated with clinical features, we performed WGCNA on GBM data from the TCGA database. The co-expression network construction revealed a soft threshold power (β) of 6, achieving a scale-free topology fit index of 0.90 (**Figure 2E**). Average linkage hierarchical clustering with this threshold identified eight distinct modules (**Figure 2F**). WGCNA generated eleven color-coded modules, excluding unassigned genes grouped in the gray module (**Figure 2G**). The module-trait relationship heatmap indicated that the red module showed the strongest correlation with clinical characteristics (**Figure 2H**). From this analysis, we extracted 674 GBM-related differential genes for further investigation.

### 3.4 TREM1 identified as a key biomarker for poor prognosis in TREM1^High^ TAM of GBM patients

**Figure 3:**
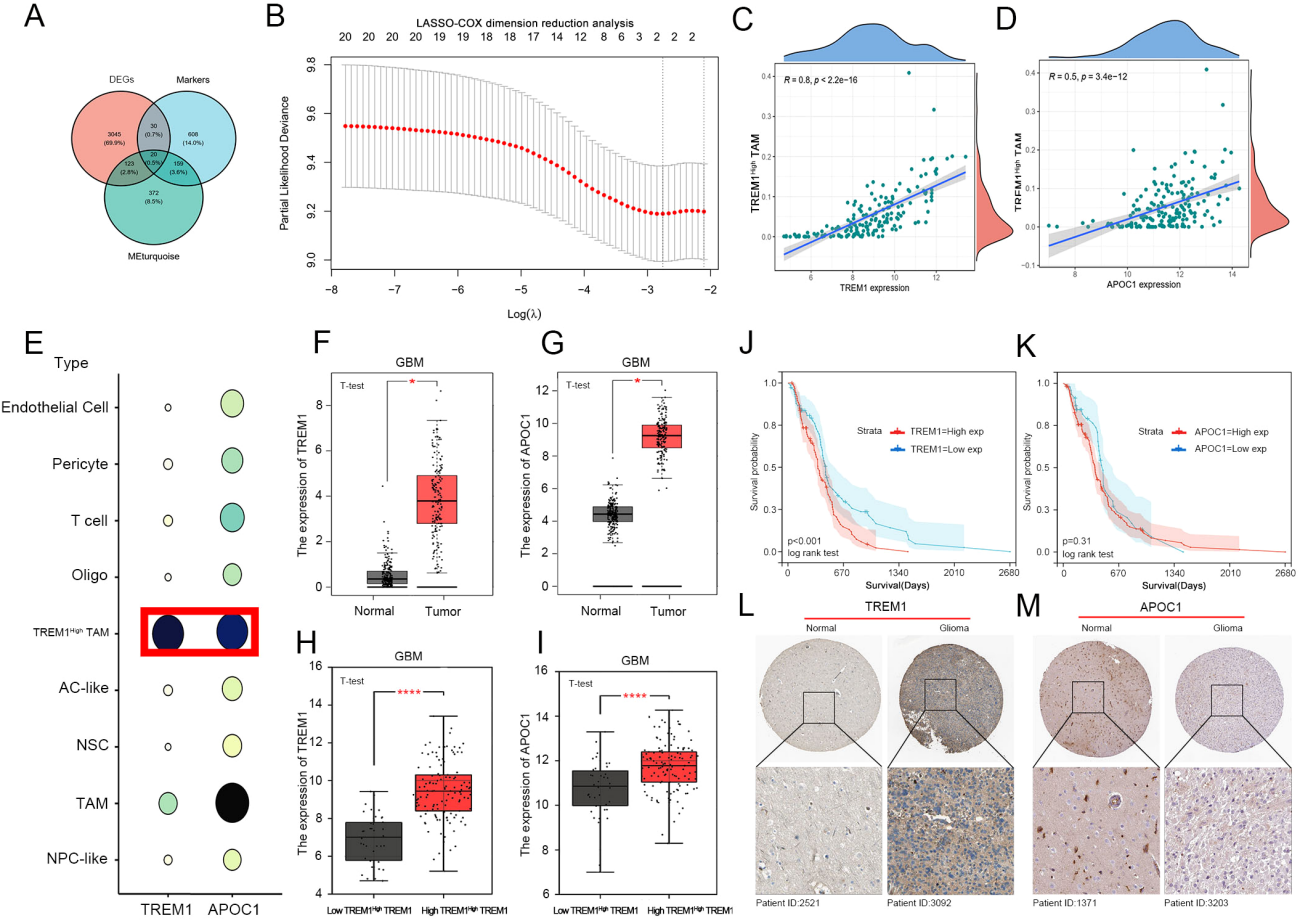
TREM1 as a Significant Biomarker for Poor Prognosis in GBM. **(A)** A Venn diagram illustrating 20 candidate biomarkers; **(B)** Lasso-Cox downscaling screening to identify 2 biomarkers associated with GBM; **(C, D)** Correlation analysis between TREM1 and APOC1 with TREM1^High^ TAM in convoluted cells; **(E)** Expression profiles of TREM1 and APOC1 derived from scRNA-seq data; **(F, G)** Differential expression of TREM1 and APOC1 between GBM and normal tissues; **(H, I)** Expression levels of TREM1 and APOC1 across different TREM1^High^ TAM subgroups within convoluted cells; **(J, K)** Kaplan-Meier curves depicting the prognostic impact of TREM1 and APOC1 in GBM patients; **(L, M)** Immunohistochemical staining demonstrating TREM1 and APOC1 expression in normal tissues and high-grade gliomas.

To further investigate the potential mechanisms underlying the association of the defined TREM1^High^ TAM with GBM clinical outcomes, intersection analysis was performed on 3218 GBM-associated differentially expressed genes (DEGs, with inclusion criteria of FC > 3 and p < 0.01), as well as 817 TREM1^High^ TAM-associated markers and 674 WGCNA trait-associated Red Module genes. This analysis identified twenty candidate biomarkers (**Figure 3A**). Considering the relationship between prognosis and gene expression, two biomarkers associated with GBM, including TREM1 and APOC1, were selected from the twenty candidate biomarkers using lasso-cox dimensionality reduction for further analysis (**Figure 3B**). Further examining the correlation between these two biomarkers and the TREM1^High^ TAM cell subtypes we identified, we found a strong correlation between TREM1 and TREM1^High^ TAM cell subtypes (r=0.8)(**Figure 3C**), while the correlation between APOC1 and TREM1^High^ TAM cell subtypes was moderate (r=0.5)(**Figure 3D**). Additionally, we reviewed the expression distribution of TREM1 and APOC1 in single-cell profiles, observing that TREM1 exhibited high expression specificity in the TREM1^High^ TAM cell subtypes we defined, whereas APOC1 showed a broadly high expression profile across virtually all defined cell subtypes (**Figure 3E**). Utilizing data from the TCGA and GTEx databases, we found that both TREM1 and APOC1 were highly expressed in GBM (**Figures 3F, G**). Immunohistochemistry results from the HPA database (HPA: http://www.proteinatlas.org/) indicated that TREM1 was highly expressed in high-grade gliomas (**Figure 3L**), in contrast to APOC1 (**Figure 3M**). Based on the TREM1^High^ TAM convolutional cell score, we observed that both TREM1 and APOC1 had high scores in the high TREM1^High^ TAM group (**Figures 3H, I**). To elucidate their prognostic characteristics, Kaplan-Meier curves (based on the optimal cutoff values) demonstrated that GBM patients with high TREM1 expression experienced a worse prognosis (**Figure 3J**), and there was no significant association between APOC1 and the prognosis of GBM patients (**Figure 3K**). Similarly, similar results were also obtained in the CGGA database (**Supplementary Figure S5**).

### 3.5 TAM-associated TREM1 inhibits GBM malignant progression

**Figure 4:**
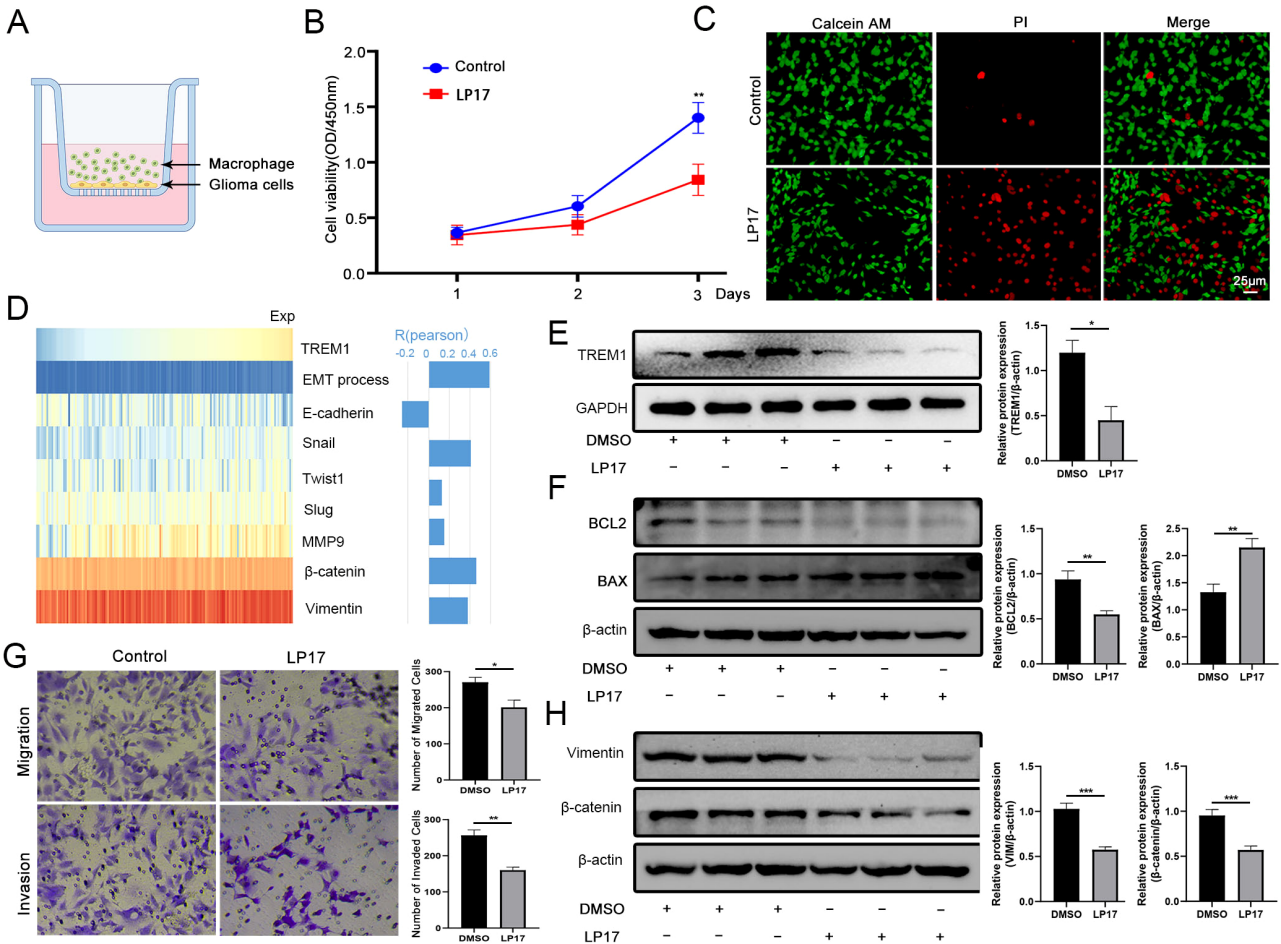
TREM1 Promotes the Malignant Progression of GBM. **(A)** Transwell experimental model diagram; **(B)** CCK-8 assay demonstrating the effect of LP17 treatment on the viability of LN229 cells; **(C)** AM/PI staining showing the viability status of LN229 cells following LP17 treatment; **(D)** Heatmap depicting the correlation between TREM1 and EMT markers; **(E)** Western blot analysis revealing the inhibitory effect of LP17 on TREM1 expression in THP1 cells; **(F)** Western blot analysis of apoptosis-related proteins in LN229 cells after LP17 treatment; **(G)** Transwell assay assessing the impact of LP17 treatment on the migratory and invasive capabilities of LN229 cells; **(H)** Western blot analysis of EMT-related markers in LN229 cells following LP17 treatment. Bar graphs represent the quantitative statistical results of the corresponding protein expression levels. Western blot results shown are representative of three independent biological replicates (n=3). *P < 0.05, **P < 0.01, ***P < 0.001.

Western blot analysis was conducted to evaluate the inhibitory effect of LP17 treatment on TREM1 expression in THP-1 cells (**Figure 4E**). The CCK8 assay demonstrated that suppression of TREM1 expression in THP-1 cells using LP17 significantly reduced the proliferative capacity of LN229 cells (**Figure 4B**). Concurrently, AM/PI staining revealed that TREM1 inhibition markedly increased the proportion of apoptotic cells in LN229 cells (**Figure 4C**). Western blot analysis further indicated that LP17 treatment upregulated the protein expression of BAX while downregulating BCL2 expression (**Figure 4F**).

Additionally, our extended bioinformatics analysis revealed a strong correlation between TREM1 expression levels in the TCGA database and the EMT process, along with associated molecular markers (**Figure 4D**). To corroborate these bioinformatics findings, we utilized the transwell co-culture model to assess the impact of LP17 on LN229 cell migration and invasion (**Figure 4A**). The results demonstrated that LP17 significantly impaired the migratory and invasive capabilities of LN229 cells (**Figure 4G**). Moreover, LP17 exhibited a pronounced inhibitory effect on EMT-promoting molecules, including Vimentin and β-catenin (**Figure 4H**).

### 3.6 TREM1 promotes the PMT process in GBM via the TLR4/PI3K/AKT/mTOR signaling axis

**Figure 5:**
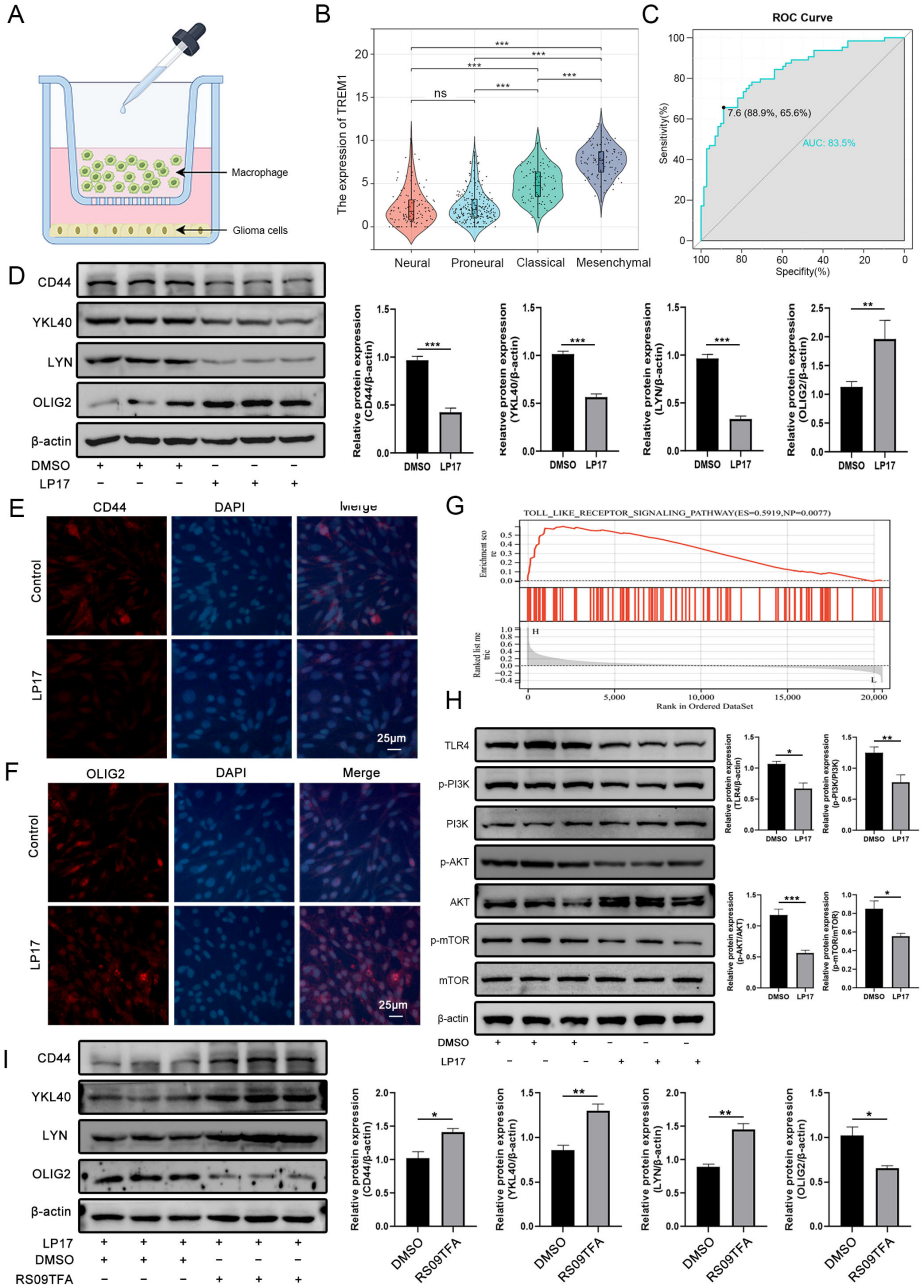
TREM1 promotes PMT in GBM cells through the TLR4/PI3K/AKT/mTOR signaling axis. **(A)** Schematic diagram of the Transwell co-culture system; **(B)** Correlation analysis between TREM1 expression and GBM molecular subtypes; **(C)** ROC curves demonstrating the specificity of TREM1 expression in the MES subtype; **(D)** Western blot analysis of protein expression changes in PN and MES subtype markers following LP17 treatment; **(E, F)** Immunofluorescence staining showing changes in CD44 and OLIG2 protein expression in LN229 cells after LP17 treatment; **(G)** GSEA revealing the potential mechanisms regulated by TREM1; **(H)** Western blot analysis of LP17 treatment effects on the activity of the TLR4/PI3K/AKT/mTOR signaling axis; I, Rescue experiments using the TLR4 agonist RS09TFA. Bar graphs represent the quantitative statistical results of the corresponding protein expression levels. Western blot results shown are representative of three independent biological replicates (n=3). *P < 0.05, **P < 0.01, ***P < 0.001.

The heterogeneity and plasticity exhibited by GBM cells are significant factors contributing to treatment resistance and relapse, while the PMT process resembles EMT seen in many aggressive cancers (26). Analysis of data from the TCGA database revealed significant enrichment of TREM1 expression in mesenchymal subtypes of GBM patients compared to other subtypes (**Figure 5B**). Additionally, the area under the ROC curve (83.5%) demonstrated the expression specificity of TREM1 in mesenchymal subtypes (**Figure 5C**). Pearson correlation analysis indicated that TREM1 positively correlates with MES subtype markers CD44, YKL40, and LYN, while showing a negative correlation with the PN subtype marker OLIG2 (**Supplementary Figure S6**). Using a Transwell co-culture model with THP-1 cells in the upper chamber and glioma cells in the lower chamber, we investigated the role of TREM1 in the PMT process of GBM (**Figure 5A**). Western blot analysis confirmed that treatment with LP17 decreased the expression of MES subtype markers CD44, YKL40, and LYN, while upregulating the expression of the PN subtype marker OLIG2 (**Figure 5D**). Immunofluorescence analysis of CD44 and OLIG2 protein expression in LN229 cells yielded consistent results (**Figures 5E, F**). To explore the potential mechanism by which TREM1 promotes PMT in GBM cells, GSEA analysis suggested that TREM1 may regulate the toll-like receptor signaling pathway (**Figure 5G**). In conjunction with the findings from functional enrichment analysis, and considering the significant role of TLR4 in regulating immune and inflammatory processes, Western blotting confirmed changes in TLR4 and its downstream PI3K/AKT/mTOR signaling axis in LN229 cells following LP17 intervention. The results indicated that LP17 treatment significantly reduced TLR4 expression levels, as well as the phosphorylation levels of p-PI3K, p-Akt, and p-mTOR (**Figure 5H**). The subsequent rescue experiments using the TLR4 agonist RS09TFA confirmed this mechanism, showing a reversal of PMT-related biomarker expression in the LP17+RS09TFA co-treatment group compared to the LP17-only group (**Figure 5I**). These findings suggest that TREM1 regulates the PMT process in GBM patients through the TLR4/PI3K/AKT/mTOR signaling axis, positioning TREM1 as a promising target for improving therapeutic outcomes in glioma patients.

### 3.7 TREM1 promotes glioma proliferation *in vivo*

**Figure 6:**
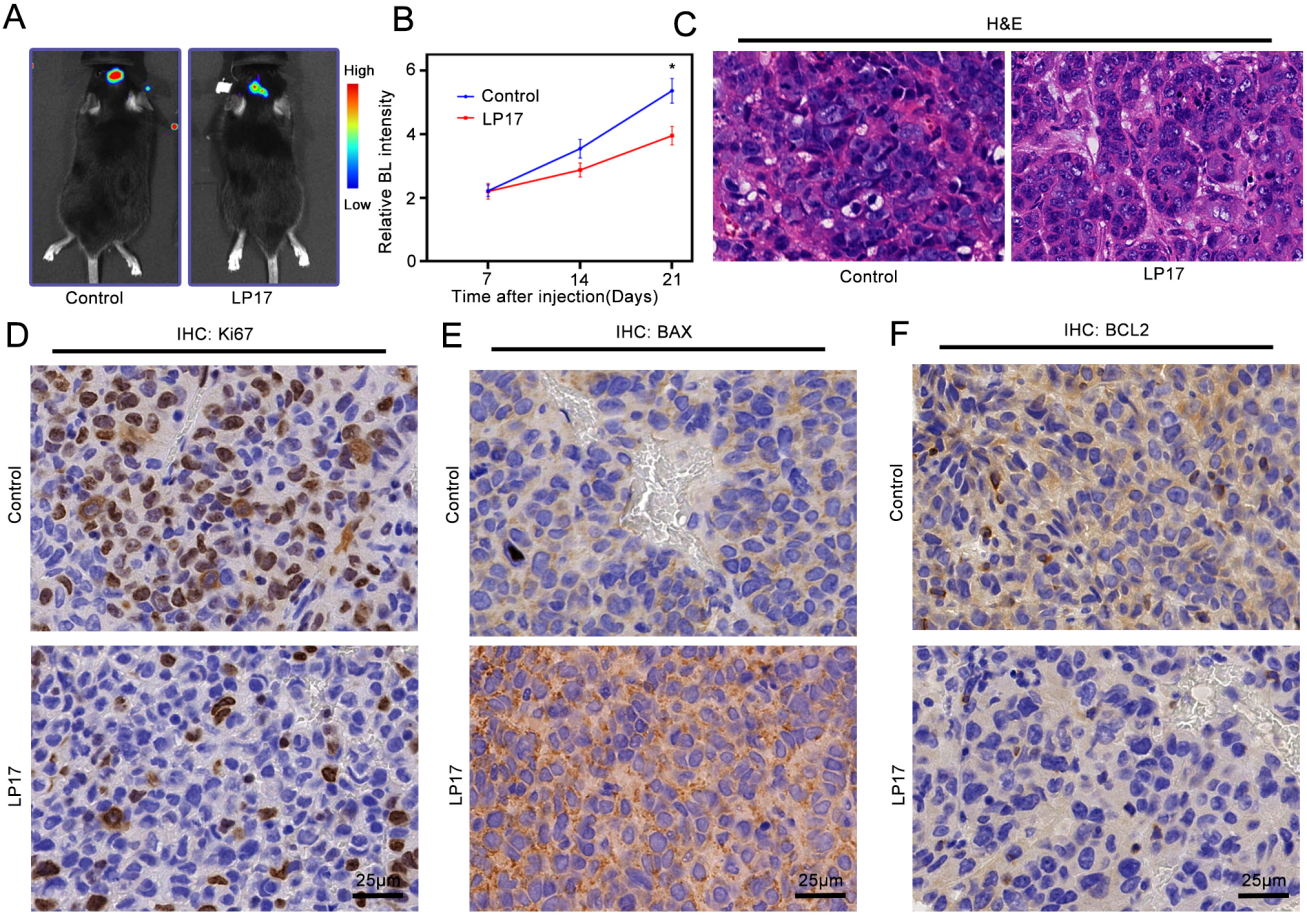
TREM1 promotes tumor cell proliferation *in vivo*. **(A)** Bioluminescence imaging to detect the effect of LP17 on tumor growth; **(B)** Bioluminescence imaging fluorescence intensity statistical graph; **(C)** Tumor H&E staining; **(D–F)** Tumor IHC staining. *P < 0.05.

To investigate the role of TREM1 in tumor progression *in vivo*, we established an orthotopic glioma model using GL261-luc cells and administered LP17 via intraperitoneal injection. Bioluminescence imaging revealed significantly slower tumor growth in LP17-treated mice compared with controls (**Figure 6A**), accompanied by reduced tumor volume (**Figure 6B**). Although HE staining showed no marked morphological differences between groups, the LP17-treated tumors exhibited lower cellular density (**Figure 6C**). Immunohistochemical analysis demonstrated decreased Ki-67 positivity in LP17-treated tumors (**Figure 6D**), consistent with the established role of this nuclear antigen as a proliferation marker in cancer diagnostics and research. Subsequent evaluation of apoptosis-related proteins showed upregulated BAX expression (**Figure 6E**) and downregulated BCL2 levels (**Figure 6F**) following LP17 treatment.

## 4 Discussion

This study offers significant insights into the role and mechanism of TREM1 in the PMT transformation in glioma patients. Earlier findings from this research highlighted the involvement of TREM1 in TAM cells during the glioma PMT process. By meticulously analyzing single-cell sequencing data in conjunction with the TCGA database, we identified that TAM cell populations with elevated TREM1 expression were correlated with a poorer prognosis in glioma patients. TREM1 was notably enriched in glioma subtypes with higher malignant potential, particularly the MES subtype. *In vitro* experiments substantiated the role of TREM1 in the proliferative and invasive processes of gliomas, demonstrating that TREM1 exerts its biological functions through the TLR4/PI3K/AKT/mTOR signaling axis, thereby facilitating the PMT transformation of glioma cells. *In vivo* inhibition of TREM1 with LP17 likewise resulted in attenuated tumor growth and enhanced tumor cell apoptosis.

Inter-and intra-tumor heterogeneity presents unprecedented challenges to targeted glioma therapy and contributes to the development of therapy resistance (27, 28). Recent studies have demonstrated that inhibiting the glioma PMT process effectively mitigates treatment resistance and reduces tumor recurrence. CD44 has been identified as a surrogate marker of the mesenchymal identity (29), while YKL40 serves as a marker for connectivity independent of the mesenchymal cell state (30). Additionally, OLIG2 has been shown to dictate glioma stem cell subtype identity, as its loss triggers a shift from the proneural transcriptional subtype to other subtypes (31). In addition, previous studies have shown that TREM1 may play a role in the immunobiological processes of glioblastoma (32). TREM1 is preferentially expressed by M2-like TAMs and induces a mesenchymal-like state in glioma stem cells (GSCs) by modulating the secretion of TGFβ2, thereby activating the TGFβR/SMAD2/3 signaling pathway in GSCs (33). Our findings reveal that TREM1 expression is closely associated with the MES subtype of gliomas. Modulating TREM1 expression effectively regulates the protein levels of PMT-related markers, including CD44, YKL40, LYN, and OLIG2. These results underscore the pivotal role of TREM1 in driving the glioma PMT process, highlighting its potential as a therapeutic target.

The hyperactivation of the PI3K/AKT pathway in tumors has emerged as a promising target for targeted cancer therapies, given its critical role in promoting cell proliferation, EMT, and metastasis (34). Recent studies have revealed that TREM1 is a cell surface receptor expressed on neutrophils, monocytes, and certain tissue macrophages, where it acts as an immunoregulator that controls myeloid cell responses (35). TREM1 in macrophages facilitates their transformation into the M1 subtype via the PI3K/AKT signaling pathway, thereby enhancing cancer cell invasiveness (36). Our bioinformatics analysis further confirmed that TREM1 exerts anti-tumor effects in gliomas by targeting TLR4. Mechanistically, TLR4 activation initiates a signaling cascade that activates various downstream effector molecules, including the PI3K/AKT pathway (37). Immunoprecipitation studies in hepatocellular carcinoma cells have demonstrated that TREM1 forms a complex with TLR4 to activate downstream signaling (38). Notably, several PI3K-targeted inhibitors are currently undergoing clinical trials (39). Our study highlights that TREM1 modulates the TLR4/PI3K/AKT/mTOR signaling axis to regulate biological functions and the PMT process in glioma cells. These findings provide valuable insights into potential alternative targets for the development of more specific and clinically effective targeted therapies. Furthermore, our rescue experiments employing the TLR4 agonist RS09TFA established that TLR4 activation can partially counteract the PMT suppression resulting from TREM1 inhibition in glioma. This finding provides direct experimental evidence confirming the pivotal role of the TREM1/TLR4/PI3K/AKT/mTOR signaling axis in regulating the PMT process in glioma.

In conclusion, our study reveals the pivotal role of TAM-associated TREM1 in regulating the PMT process in GBM and elucidates the underlying mechanism by which TREM1 drives GBM progression. The prominent expression of TREM1 in the MES subtype of GBM underscores its significant biological relevance in GBM pathogenesis. *In vitro* experiments demonstrated that targeted inhibition of TREM1 not only exerted anti-tumor effects but also suppressed the PMT process in GBM and inhibited the activation of the TLR4/PI3K/AKT/mTOR signaling axis, further highlighting its clinical significance in GBM progression. These findings provide a valuable foundation for the development of targeted therapies against GBM.

These findings provide a valuable foundation for the development of targeted therapies against GBM. However, further in-depth studies are required to fully elucidate the intricate mechanisms of TREM1-mediated signaling between TAMs and tumor cells. As suggested by prior work in other cancers, a direct physical interaction between TREM1 and TLR4 may underpin the observed signaling activation. To explicitly test this in our context, future work will employ co-immunoprecipitation (Co-IP) and proximity ligation assays (PLA) in primary GBM-derived TAMs and co-culture systems to confirm the formation of a TREM1/TLR4 complex. Furthermore, utilizing TREM1-knockout macrophage models would help delineate the specific contribution of TREM1 in initiating this interaction and activating the downstream TLR4/PI3K/AKT/mTOR axis, thereby providing direct mechanistic validation of our current findings.

## 5 Conclusion

In this study, we elucidated the expression pattern, molecular biological functions, and underlying mechanisms of TREM1 in GBM patients through single-cell sequencing analysis. Our findings demonstrate that TREM1 promotes the PMT process in GBM and modulates the TLR4/PI3K/AKT/mTOR signaling axis. These results provide critical insights for clinically inhibiting GBM progression and developing targeted therapeutic strategies.

## Data Availability

The datasets presented in this study can be found in online repositories. The names of the repository/repositories and accession number(s) can be found below: https://www.ncbi.nlm.nih.gov/geo/, GSE135045.
