# Supplementary material for: Unveiling the novel value of TREM1 in the proneural-mesenchymal transition of glioma via tumor-associated macrophages
Source: Front Immunol. 2025 Nov 27;16:1662351. doi: 10.3389/fimmu.2025.1662351 (PMC12695795; doi:10.3389/fimmu.2025.1662351)
Supplement: Supplementary file 1 [file Presentation1.pdf]

Supplementary documents

Title Page

Article title:

Unveiling the novel value of TREM1 in the proneural-mesenchymal transition of glioma via tumour - associated macrophages

Authors' names:

Chao Zhang[1#], Ben Hu[2#], Chao Wang[1#], Shiqiang Hou[1]*, Ning Lin[1]*

Authors' affiliations:

[1]Department of Neurosurgery, The Affliated Chuzhou Hospital of Anhui Medical University, The First People's Hospital of Chuzhou, Chuzhou 239000, China.

[2]Science and Technology Innovation Center, Guangzhou University of Chinese Medicine, Guangzhou 510405, China.

Corresponding author:

Ning Lin: linning@ahmu.edu.cn

Shiqiang Hou: houshiqiang@ahmu.edu.cn

Supplementary Figure S1

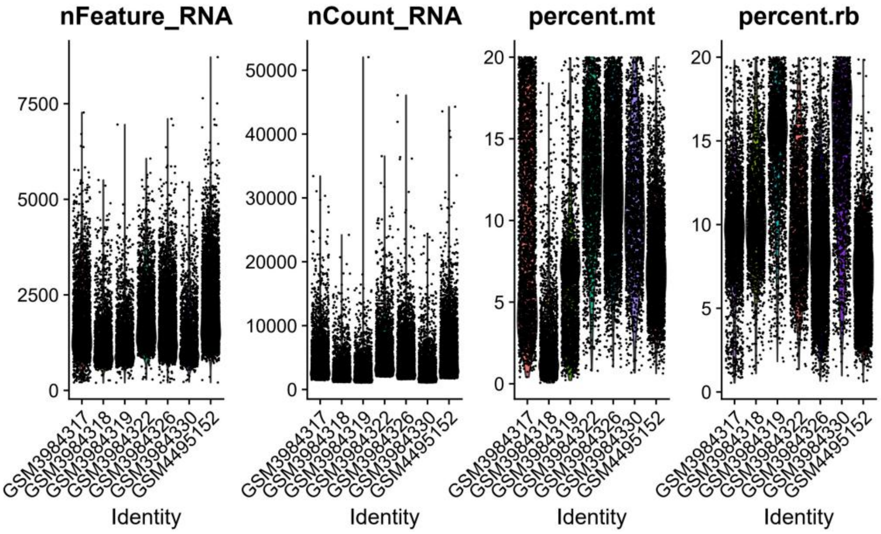

Supplementary Figure S1: Quality Control Criteria for Single-Cell RNA-seq (scRNA-seq) Data: Analyzing the distribution of key metrics, including nFeature_RNA, nCount_RNA, percent.mt, and percent.rb.

Supplementary Figure S2

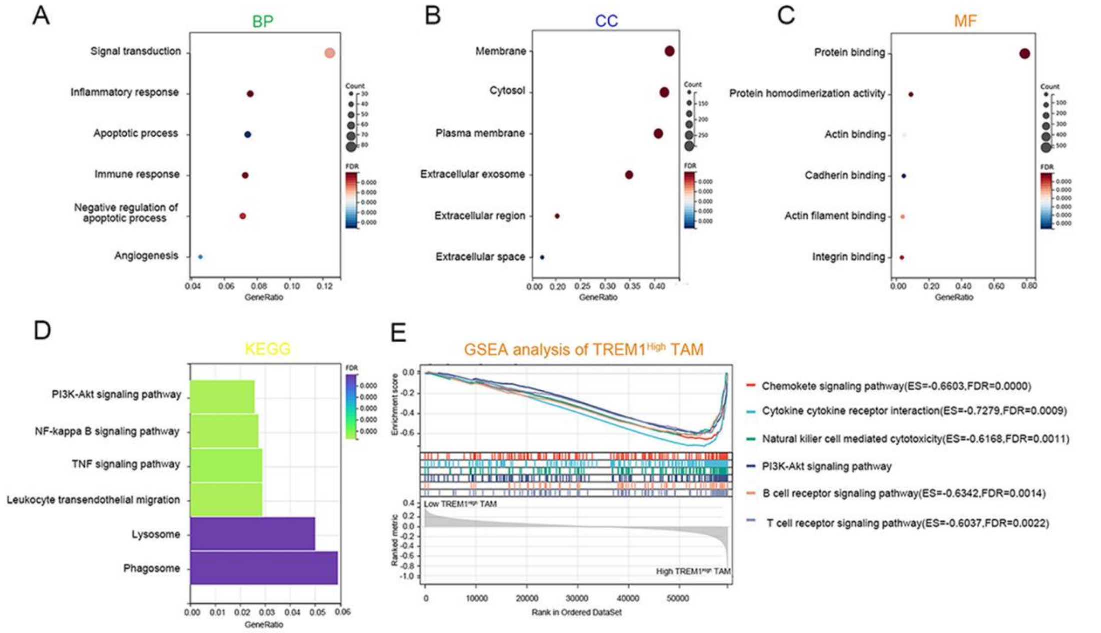

Supplementary Figure S2: Functional Enrichment Analysis Pertaining to Convoluted Cell TREM1^{High} TAM. A, Outcomes of GO Analysis for Convoluted Cell TREM1^{High} TAM Genes in Biological Process (BP); B, Outcomes of GO Analysis for Convoluted Cell TREM1^{High} TAM Genes in Cellular Component (CC); C, Outcomes of GO Analysis for Convoluted Cell TREM1^{High} TAM Genes in Molecular Function (MF); D, Results of KEGG Analysis for Convoluted Cell TREM1^{High} TAM Genes; E, GSEA Analysis Results for Convoluted Cell TREM1^{High} TAM.

Supplementary Figure S3

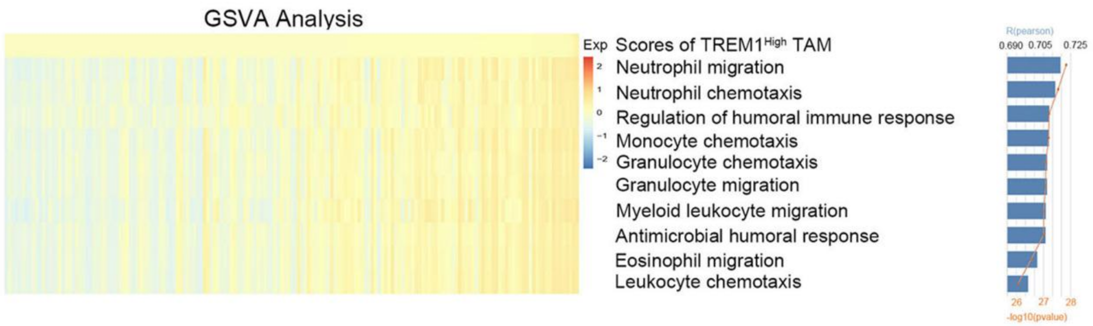

Supplementary Figure S3: GSVA analysis of convoluted cell TREM1^{High} TAM-related functions.

Supplementary Figure S4

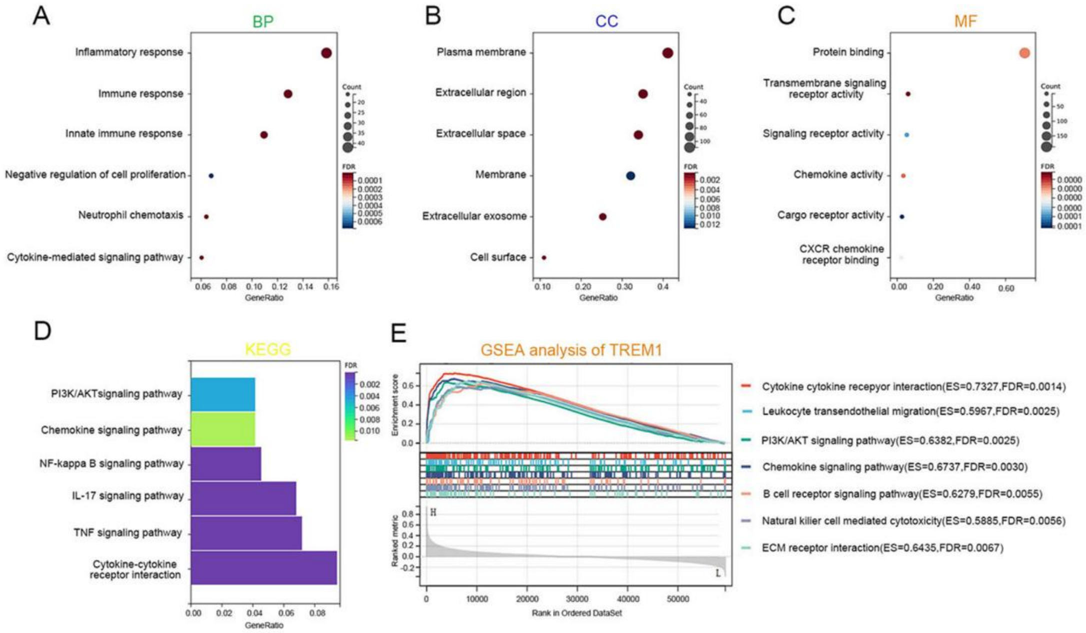

Supplementary Figure S4: Functional enrichment analysis of TREM1 co-expressed genes.A, Biological process (BP); B, Cellular component (CC); C, Molecular function (MF); D, KEGG analysis results; E, GSEA analysis results.

Supplementary Figure S5

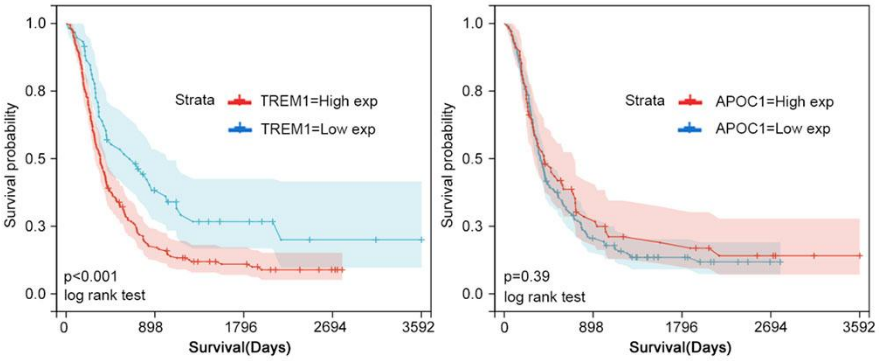

Supplementary Figure S5: Kaplan-Meier curves depicting the prognostic impact of TREM1 and APOC1 in CGGA database of GBM patients.

Supplementary Figure S6

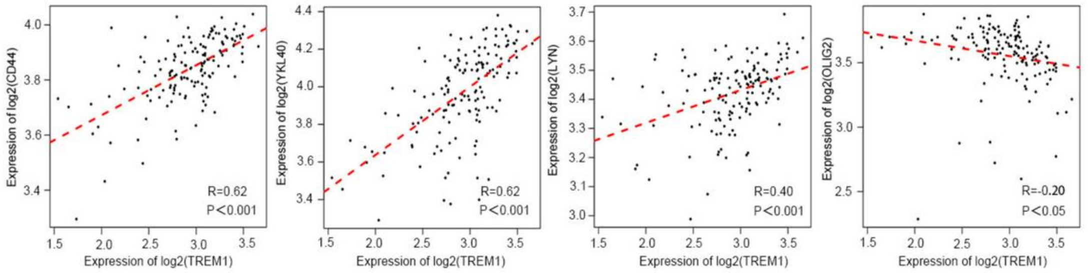

Supplementary Figure S5: Pearson correlation analysis of TREM1 expression with PN and MES subtype markers.
